# Cholinergic and oxidative stress mechanisms in sudden infant death syndrome

**DOI:** 10.1111/j.1651-2227.2009.01476.x

**Published:** 2009-11

**Authors:** Anne Dick, Rodney Ford

**Affiliations:** 1 Canterbury Cot Death Fellow, Community Paediatric UnitChristchurch, New Zealand; 2 Consultant Paediatrician, Children’s Gastroenterology and Allergy ClinicChristchurch, New Zealand

**Keywords:** Cholinergic, Oxidative stress, Sudden infant death syndrome

## Abstract

**Aim:**

To determine whether biochemical parameters of cholinergic and oxidative stress function including red cell acetylcholinesterase (AChE), serum/plasma thyroglobulin, selenium, iron, ferritin, vitamins C, E, and A affect risk in apparent life-threatening event (ALTE), sudden infant death syndrome (SIDS), and sudden unexpected death in infancy (SUDI). To assess these biochemical parameters as a function of age; and for influence of pharmacology and epidemiology, including infant health, care, and feeding practices.

**Methods:**

A multicentre, case–control study with blood samples from 34 ALTE and 67 non-ALTE (control) infants matched for age, and 30 SIDS/SUDI and four non-SIDS/non-SUDI (post-mortem control) infants.

**Results:**

Levels/activity of the biochemical parameters were not significantly different in ALTE vs. control infants, with the exception of higher vitamin C levels in the ALTE group (p = 0.009). In ALTE and control groups, AChE and thyroglobulin levels increased and decreased respectively from birth to attain normal adult levels from 6 months. Levels of iron and ferritin were higher in the first 6 month period for all infant groups studied, intersecting with vitamin C levels peaking around 4 months of age.

**Conclusion:**

Lower AChE levels and higher combined levels of iron and vitamin C in the first 6 months of life may augment cholinergic and oxidative stress effect, particularly at the age when SIDS is most prevalent. This may contribute to risk of ALTE and SIDS/SUDI events during infancy.

## Introduction

A unifying mechanism for sudden infant death syndrome (SIDS) remains elusive. It is widely accepted, however, that SIDS infants have a vulnerable predisposition, exacerbated by exogenous stressors, at a critical stage of development (1). Against this background the sleep state, often with compromised sleep situation, appears to determine whether SIDS is the outcome or not.

As a possible mechanism for SIDS, this study proposed that increased cholinergic tone and oxidative stress contribute to vulnerability in SIDS infants, with vagally mediated reflexes and oxidative stressors exacerbating this vulnerability, most critically in a sleep state affected by hypoxia.

The rationale for this mechanism, and the biochemical parameters examined, reflect known epidemiological, clinical, and pathological features of the syndrome. Cholinergic transmission, acting centrally; and peripherally at autonomic ganglia, parasympathetic effector organs, and neuromuscular junctions; can be increased directly (receptor agonists), through mimicking of the acetylcholine neurotransmitter e.g. nicotine, or indirectly by inhibiting the enzyme acetylcholinesterase (AChE), which degrades the neurotransmitter (2). Increased cholinergic activity producing mucus secretion, sweating, vomiting, sphincter relaxation, airway obstruction, bradycardia, and initial muscle tonicity followed by more prolonged flaccidity, has been commonly reported in apparent life-threatening event (ALTE) infants, and SIDS infants prior to their deaths (3,4).

Reactive oxygen species or free radicals are constantly produced in the body, with protection of body tissues from oxidative stress conferred by antioxidants (5). Intermittent hypoxia then reoxygenation, as experienced premorbidly by some SIDS infants, and oxidation of catecholamines produced in response to stress, can generate superoxide free radicals (6). While superoxide itself is relatively unreactive, it acts as a precursor for more reactive species with its conversion to hydrogen peroxide, which in turn may be converted in the presence of transition metal ions e.g. Cu^+^ and Fe^++^ (vitamin C acting as an oxidant reducing Fe^+++^ to Fe^++^) to the most reactive and damaging of the oxygen free radicals, the hydroxyl (Fenton reaction) (5,6).

One of the intracellular targets for the hydroxyl radical is the phospholipid component of membranes resulting in lipid peroxidation and membrane disruption (5). In the blood vessels and mucosa of the respiratory tract, lipid peroxidation combined with oxidative release of inflammatory mediators (7) and vagally mediated neurogenic inflammation (8), may produce the intrathoracic petechiae, pulmonary congestion and oedema, and inflammatory infiltrate often seen in SIDS victims (9).

Vitamin E, as alpha-tocopherol, acts as a chain breaking antioxidant in lipid peroxidation intercepting lipid peroxyl radicals. Vitamin C reacts with the tocopherol radical produced to reconvert it to alpha-tocopherol, thereby acting as an antioxidant in the absence of transition metal ions rather than an oxidant (5).

The provitamin A carotenoid beta-carotene is also a scavenger of peroxyl radicals. This activity may be increased in the presence of other antioxidants, e.g. vitamin E, but pro-oxidant activity has been reported when taken in high dose and in the presence of highly oxidative conditions (10).

Selenium is involved in oxidative protection as an essential component of glutathione peroxidase, which safely reduces hydrogen peroxide and other organic hydroperoxides (5,6).

A possible relationship between AChE and thyroglobulin [precursor of triiodothyronine (T3) and tetraiodothyronine (T4) thyroid hormones], is reflected in their shared homologies, immunoreactivity, and oxidative chemistry. The Fenton chemistry, which cleaves thyroglobulin within its cholinesterase-like-domain into immunoreactive, T3, T4 hormone producing peptides (11), has also been shown to cleave and deactivate AChE (12).

The aims of this study were therefore: (i) to assess if activity of AChE, reflecting the enzyme AChE that inactivates acetylcholine in the cholinergic nervous system, was low in ALTE, SIDS, and sudden unexpected death in infancy (SUDI) infants; (ii) to evaluate thyroglobulin for reciprocal inverse response with AChE; (iii) to assess if decreased oxidative stress protection, as determined by low levels of antioxidants selenium, vitamin E, ± vitamin A, high levels of pro-oxidants iron and ferritin, and conditional pro-oxidant effect of vitamin C, was present in ALTE and SIDS/SUDI infants; (iv) to measure levels/activity of these biochemical factors as a function of age to determine if they confer influence on the age of risk for SIDS; and (v) to examine the influence of pharmacology and epidemiology, including infant health, care, and feeding practices, on these biochemical factors.

## Methods

This multicentre, case–control study was conducted in New Zealand, at Auckland, Wellington, Christchurch, and Dunedin hospitals, over a 10-year period between 1996 and 2006. After obtaining informed consent from parents or guardians, blood samples of up to 3.5 mL were collected from ALTE and non-ALTE (control) infants, and SIDS/SUDI and non-SIDS/non-SUDI (post-mortem control) infants. Attempt was made to perform all biochemical assays on samples collected, i.e. AChE, also measured per gram haemoglobin (AChE/gHb) recognizing changing red cell mass with infant age, serum/plasma thyroglobulin, selenium, iron, ferritin, and vitamins C, E, and A.

Blood samples from ALTE and control infants matched for age, two for each case, were obtained between 1996 and 2006 at Christchurch Hospital. The additional blood sample was taken at the time of blood sampling for other reasons, in the medical management of these infants. Inclusion criteria for ALTE infants were a frightening event, between 1 week and 1 year of age, characterized by at least two out of three of the following: (i) apnoea (central or occasionally obstructive), choking or gagging; (ii) colour change (usually cyanotic or pallid, but occasionally erythematous or plethoric); and (iii) marked change in muscle tone (usually marked limpness) (13). Infants were excluded from ALTE and control groups if tonic-clonic seizure was a feature of their presentation.

Blood samples were collected from the superior vena cava for SIDS/SUDI and post-mortem control infants presenting for post-mortem between 1996 and 2006. All SIDS/SUDI samples were collected at Christchurch Hospital. With only small numbers of non-SIDS/non-SUDI infants presenting, local ethics approval was sought to extend the study to hospitals in Dunedin in 1996, and Auckland and Wellington in 1997, for the post-mortem collection of control samples. In this study, the 1969 definition of SIDS was used: ‘the sudden death of an infant or young child, which is unexpected by history, and in which a thorough post-mortem examination fails to demonstrate an adequate cause for death’ (9). From 1998, an upper limit of 1 year was set in accordance with local practices for SIDS classification. For the purposes of this study, infants under 1 year satisfying the 1969 SIDS definition were classified as SIDS, and a classification of SUDI was given to infants with similarly unexplained deaths aged between 1 and 2 years. Causes of death and their classification were reviewed by the pathologist who conducted the majority of post-mortems over the study period.

To examine the influence of pharmacology and epidemiology on these biochemical factors, a questionnaire was completed with reference to obstetric, medical, and nursing records of the infant.

Blood samples were analysed by Canterbury Health Laboratories, the Free Radical Research Group (University of Otago), and LabPlus Auckland. Over the course of the study, assay methods changed, but correlation assays confirmed no significant effect on the consistency of measurement. Changes in method standardization for the AChE assay, however, did affect reported results. To ensure consistent measurement, adjusted AChE and AChE/gHb levels were used, calculated from results for all AChE assays performed at the laboratory during the study period. There were also concerns over the validity of vitamin C assays reported from two batches and these were excluded from analyses.

Assay methods for this study included the following. For AChE, a colorimetric determination was performed using the 5,5-dithiobis-2-nitrobenzoate (DTNB) method for the Cobas Bio analyser (Roche Diagnostics, Indianapolis, IN, USA). Haemoglobin measurement for AChE/gHb was performed using Coulter technology, a Coulter STKS analyser (Coulter, Hialeah, FL, USA) until 1999, and a Coulter GenS analyser (Coulter Corp., Miami, FL, USA), thereafter. Thyroglobulin assays were performed using the Sorin (Sorin Biomedica Diagnostics S.p.A., Saluggia, Italy) immunoradiometric (IRMA) method until the end of October 2002, an automated method using the Immulite analyser (Siemens, Tarrytown, NY, USA) from November 2002, and a chemiluminescent immunoenzymatic assay on the Access analyser (Beckman Coulter Inc., Fullerton, CA, USA) from May 2003. Plasma selenium was measured by graphite furnace atomic absorption spectroscopy using pyrolytically coated graphite partition tubes and Zeeman background on a Varian 220Z analyser (Varian Inc., Mulgrave, Australia). The iron assay was performed using photometric measurement, initially with Roche reagents (Roche Diagnostics GmbH, Mannheim, Germany) on the Hitachi 717 (Roche Diagnostics), and later with Sentinel reagents (Sentinel Diagnostics, Milan, Italy) on the Abbott Aeroset and Abbott Architect c8000 analysers (Abbott Laboratories, Abbott Part, IL, USA). The IMX ferritin assay (Abbott Laboratories), based on the microparticle enzyme immunoassay (MEIA) technology, was used until the end of April 2005, and was then replaced with the Architect ferritin assay (Abbott Laboratories), a chemiluminescent microparticle immunoassay (CMIA). Plasma vitamin C was measured by high performance liquid chromatography (HPLC) using a C18 column (Phenomenex, San Jose, CA, USA) with electrochemical detection. Vitamins E and A were similarly measured by HPLC using a C18 column (Phenomenex) with ultraviolet detection.

The statistical analysis system (sas) version 9.1 (SAS Institute Inc., Cary, NC, USA) was used for statistical analysis. ALTE and control groups were analysed using *t*-tests for paired samples. Biochemical parameters for each ALTE infant were compared with mean biochemical parameters from available controls for that infant. Correlations and partial correlations were used to analyse the relationships between AChE, AChE/gHb, thyroglobulin, and age; analysis of variance (ANOVA) and analysis of simple effects, the relationship between smoking and thyroglobulin levels. To examine the relationships between iron/ferritin levels, infant age, and method of feeding, regression analysis was carried out to decide terms for age in the model, with breast-fed and formula-fed infants compared using ANOVA. The frequency of maternal iron supplementation and influence on iron/ferritin levels in the infant groups were examined using chi-square and pooled *t*-tests respectively. Logarithmic transformation was used as necessary to manage skewed data and large values for thyroglobulin, iron, ferritin, and vitamin C parameters.

Ethical approval for this study was obtained from the Canterbury Ethics Committee/Upper South Regional Ethics Committees, and the local ethics committees of other participating sites.

## Results

Blood samples were collected from 37 ALTE infants and 69 control cases. Three infants under 1 week of age were excluded from the ALTE group, and two infants presenting with tonic-clonic seizure from the control group, leaving 34 ALTE infants and 67 control infants in the study. The five infants excluded from these groups were categorized as ‘infant other’.

Post-mortem blood samples were collected from 28 SIDS infants, two SUDI, and four post-mortem control infants between 1996 and 2006. Causes of death for the four postmortem control infants were drowning, accidental asphyxia, smoke and fume inhalation, and cerebral infarction during cardiac surgery.

A comparison of biochemical parameter means for the ALTE and control groups is shown in Table 1. The only significant difference identified was higher vitamin C levels in the ALTE group (p = 0.009).

**Table 1:** Comparison of biochemical parameter means for ALTE and control infants

Parameters	n	ALTE mean (SD)	Control mean (SD)	Mean diff (SD)	95% CI	p-value
AChE (KU/L)	30	8.8 (2.2)	9.2 (2.0)	-0.4 (2.2)	−1.2 to 0.4	0.35
AChE (U/gHb)	22	34.0 (8.0)	35.8 (6.8)	-1.8 (8.4)	−5.5 to 1.9	0.32
Thyroglobulin (mcg/L)	27	44.7 (25.3)	40.5 (24.2)	4.2 (38.0)	−10.8 to 19.3	0.57
Selenium (mcmol/L)	30	0.45 (0.17)	0.47 (0.23)	-0.02 (0.35)	−0.15 to 0.11	0.71
Iron (mcmol/L)	28	10.6 (4.1)	10.8 (6.8)	-0.2 (6.5)	−2.8 to 2.3	0.84
Ferritin (mcg/L)	31	139.8 (96.8)	159.4 (109.0)	-19.6 (97.7)	−55.4 to 16.3	0.27
Vitamin C (mcmol/L)	19	81.8 (23.8)	63.3 (26.6)	18.5 (27.4)	5.3 to 31.8	0.009
Vitamin E (mcmol/L)	30	23.5 (9.6)	23.4 (10.0)	0.1 (16.0)	−5.9 to 6.1	0.97
Vitamin A (mcg/L)	30	295.9 (132.5)	261.6 (97.5)	34.2 (140.0)	−18.0 to 86.5	0.19

n = number of ALTE infants with at least one control infant; ALTE = apparent life-threatening event; AChE = red cell acetylcholinesterase; Hb = haemoglobin; SD = standard deviation; diff = difference; CI = confidence interval.

The small number of post-mortem control infants available to this study prevented case–control analysis of SIDS/SUDI infant biochemistry. Table 2 presents a summary of data for post-mortem infant groups.

**Table 2:** Post-mortem infant group data for biochemical parameters

Parameter	Infant group	n	Mean age (weeks)	Mean value	Median value	Range	Reference range
AChE (KU/L)	SIDS	28	15.3	8.0	7.8	5.1–10.8	>8
	SUDI	2	80.9	6.6	6.6	5.2–7.9	
	P-m control	3	62.1	9.4	9.8	8.5–9.9	
AChE/gHb (U/gHb)	SIDS	27	15.5	29.9	30.0	18.8–45.7	30–51*
	SUDI	2	80.9	21.7	21.7	17.6–25.7	
	P-m control	2	71.4	35.9	35.9	34.6–37.1	
Thyroglobulin (mcg/L)	SIDS	27	15.7	1004.4	358.0	34.0–5260.0	0–58
	SUDI	2	80.9	2037.5	2037.5	71.0–4004.0	
	P-m control	4	52.6	168.3	123.0	35.0–392.0	
Selenium (mcmol/L)	SIDS	27	14.6	0.48	0.45	0.11–1.08	0.45–1.40
	SUDI	2	80.9	1.02	1.02	0.64–1.39	
	P-m control	2	71.4	0.42	0.42	0.39–0.44	
Iron (mcmol/L)	SIDS	26	14.6	85.0	45.0	20.0–750.0	10–30
	SUDI	2	80.9	22.0	22.0	14.0–30.0	
	P-m control	2	71.4	17.5	17.5	11.0–24.0	
Ferritin (mcg/L)	SIDS	21	15.7	39455.7	21384.0	695.0–99000.0	15–150
	SUDI	2	80.9	1337.5	1337.5	1119.0–1556.0	(0–14 years)
	P-m control	2	71.4	1216.0	1216.0	1030.0–1402.0	
Vitamin C (mcmol/L)	SIDS	20	15.0	218.5	223.3	6.9–649.4	26–85
	SUDI	1	88.7	411.5	411.5		
	P-m control	1	90.9	95.2	95.2		
Vitamin E (mcmol/L)	SIDS	24	15.2	27.6	23.0	3.0–65.0	23–70
	SUDI	1	88.7	21.0	21.0		(rec level)
	P-m control	3	62.1	19.3	20.0	17.0–21.0	
Vitamin A (mcg/L)	SIDS	24	15.2	344.5	339.5	88.0–634.0	200–800
	SUDI	1	88.7	308.0	308.0		(rec level)
	P-m control	3	62.1	376.7	253.0	183.0–694.0	

Reference ranges provided by Canterbury Health Laboratories unless otherwise indicated.

AChE = red cell acetylcholinesterase; Hb = haemoglobin; SIDS = sudden infant death syndrome; SUDI = sudden unexpected death in infancy; P-m = post-mortem; rec = recommended.

* Sanz et al. (14).

**Figure 1:**
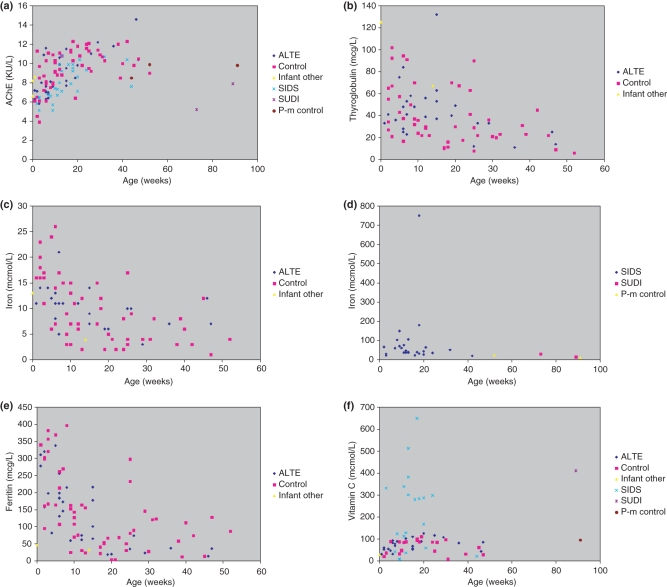
Level of biochemical parameters (a) red cell acetylcholinesterase (normal range >8); (b) thyroglobulin (reference range 0–58 mcg/L); (c) iron (reference range 10–30 mcmol/L); (d) iron post-mortem; (e) ferritin (reference range for 0–14 years, 15–150 mcg/L); (f) vitamin C (reference range 26–85 mcmol/L); as a function of age. Reference ranges provided by Canterbury Health Laboratories. AChE = red cell acetylcholinesterase; ALTE = apparent life-threatening event; SIDS = sudden infant death syndrome; SUDI = sudden unexpected death in infancy; P-m = post-mortem.

Further examination of individual biochemical parameters showed AChE and AChE/gHb to be highly correlated statistically (0.88), increasing with age 0.59/0.59 to achieve normal adult levels by 6 months of age for ALTE and control infants (Fig. 1a). Mean levels in the SIDS/SUDI infants (Table 2) were slightly lower than other infant groups, however the small numbers of post-mortem control infants and age disparity did not allow for statistical analysis.

Thyroglobulin levels reduced from birth to attain normal adult levels by 6 months of age for ALTE and control infants (Fig. 1b). In these infants statistical analysis showed AChE levels to be negatively correlated with thyroglobulin (−0.23) which in turn declined with age (−0.41). Partialling out the effect of age, there was no correlation remaining between AChE and thyroglobulin (−0.07). In SIDS/SUDI and most post-mortem control infants, levels were elevated with no apparent relationship to age or AChE levels. We have previously shown higher thyroglobulin levels in cord blood samples where there was maternal smoking (A. Dick and R. Ford, unpubl. data), with analysis of maternal smoking/non-smoking during pregnancy showing significant interaction of thyroglobulin with smoking (p = 0.01). The same interaction of thyroglobulin with maternal smoking during pregnancy (closely correlated with postnatal rate of smoking) did not extend postnatally to ALTE/control infants examined in this study (p = 0.15).

Mean selenium levels remained low normal or below normal adult levels throughout the first year (Tables 1 and 2). Method of feeding was not observed to affect selenium levels overall, however the highest levels of >1.0 mcmol/L were reported in infants on formula feeds ± solids.

Iron levels in ALTE and control infants reduced from birth to low normal adult levels from 6 months, remaining at that level during the first year of life (Fig. 1c). Mean iron levels in SIDS infants were elevated, peaking at 18 weeks with a level of >750 mcmol/L (given as 750 mcmol/L for the purposes of statistical analyses, graphs, and tables), and reducing subsequently (Fig. 1d). In ALTE and control infants, iron levels appeared to be higher in breast-fed infants, but statistical analysis comparing ‘mainly breast-fed’ and ‘mainly formula-fed’ infants (post-mortem groups and infants on solids excluded) showed this to be related to the younger age of breast-fed infants and not method of feeding (p = 0.57). Maternal iron supplementation during pregnancy (not inclusive of low-dose iron from multivitamin and mineral supplementation) was significantly higher in mothers of SIDS infants when compared with mothers of ALTE (p = 0.04) and control infants (p = 0.03). Anaemia of pregnancy was not over-represented in mothers of SIDS infants with four mothers from the ALTE group, five from the control group, and three from the SIDS group receiving iron supplementation for antenatal haemoglobins of <100 g/L. In the small numbers examined, higher mean iron levels were reported in SIDS infants (infants on iron supplementation excluded) where maternal iron supplementation occurred during pregnancy, 104 mcmol/L (n = 18) compared with 45 mcmol/L (n = 7) where no maternal iron supplementation took place. This approached significance for SIDS infants (p = 0.09), but was not evident with iron supplementation during pregnancy in other groups.

Ferritin levels increased in the first 8 weeks of life for ALTE and control infants, reducing to normal childhood levels from 6 months of age (Fig. 1e). In SIDS, SUDI, and post-mortem control infants, ferritin levels were elevated (Table 2), particularly in the SIDS group where levels ranged from 695 to >99 000 mcg/L (given as 99 000 mcg/L for the purposes of statistical analyses and tables). Six other ferritin levels, which either exceeded the upper limit for the assay at the time (reduced from >99 000 to >40 000 mcg/L in 2005), or where there was insufficient sample after repeated dilution to achieve a result, were excluded from analyses. Higher ferritin levels were also seen in breast-fed ALTE and control infants, analysis again showing this to be related to the younger age of breast-fed infants, with a non-significant trend for lower ferritin in the ‘mainly formula-fed’ group (p = 0.11). There was no statistical association observed between maternal iron supplementation during pregnancy and infant ferritin levels, although reduced ferritin data in the SIDS group may have compromised this analysis. Infant iron supplementation (by prescription only for infants in this study) did not appear to influence iron or ferritin levels for these particular infants.

Vitamin C levels for ALTE and control infants tended to be low normal adult levels in the first 3 weeks of life, increasing to maximal levels around 4 months of age, and then reducing to normal adult levels from 30 weeks of age (Fig. 1f). Levels between 6 and 30 weeks of age often exceeded the adult upper limit for vitamin C, particularly in SIDS infants, although very low levels of <10 mcmol/L were also reported in two SIDS infants.

For all the infant groups, mean vitamin E and A levels remained low or low normal when compared with recommended levels, over the first 2 years of life (Tables 1 and 2), with addition of solids appearing to increase vitamin A levels slightly.

There were trends for maternal supplementation during pregnancy with vitamins C, E, and A to increase reported levels of those vitamins in SIDS infants, and for infant supplementation with Vitadol C (Nutricia Ltd, Auckland, New Zealand) (daily dose: vitamin A 666.7 mcg, vitamin C 33.3 mg, vitamin D 11.7 mcg) to increase vitamin C levels in supplemented infants, although numbers were too small for statistical analyses. Maternal supplementing with iron and vitamins while breast feeding, was not shown to influence infant levels in any of the groups studied.

When pharmacological factors were analysed, levels of exposure to maternal smoking, alcohol, recreational drug use (e.g. marijuana, benzodiazepines), methadone, and caffeine consumption were comparable in ALTE and control groups, but at least twice the level of exposure was observed in SIDS infants compared with other groups. Use of these substances did not appear to affect the biochemical parameters studied in this study.

Opiates in some form (morphine, pholcodine, and herbal withania root) were more commonly administered in SIDS infants (14%). Infant use of these opiates, again, did not appear to influence the biochemical parameters studied, nor did maternal or infant use of any other medications examined in this study.

All SIDS and SUDI deaths occurred during assumed sleep, with 59% of ALTE infants also thought to be sleeping at the time of their events. Compromised sleep situation distinguished the SIDS/SUDI group from the ALTE group.

When infant health was examined, gastro-oesophageal reflux (GOR) was more prevalent among ALTE infants (56%), and upper respiratory symptoms among SIDS/SUDI infants (53%), although five further SIDS infants experienced spilling and unexplained irritability, which may have represented undiagnosed GOR.

No trends were observed in levels of biochemical parameters as a result of sleep situation, underbedding (with specific reference to acetycholinesterase levels), ethnicity (Maori vs. non-Maori), or the health conditions examined.

## Discussion

We have hypothesized that increased cholinergic tone and oxidative stress contribute to an increased risk of SIDS examining parameters of cholinergic and oxidative stress function.

Assessing the aims of this study, we have observed that mean AChE levels were slightly lower in the SIDS/SUDI infants compared with other infant groups, but have shown no statistical difference in AChE levels between ALTE and control infants. While thyroglobulin showed a reciprocal inverse relationship with AChE in ALTE and control infants, this was confirmed statistically to be a function of age with no direct correlation between AChE and thyroglobulin levels. Pro-oxidant parameters, iron and ferritin were elevated in SIDS infants, with ferritin levels in SUDI infants elevated to a lesser degree, comparable to levels in post-mortem control infants. Vitamin C with antioxidant properties, but also pro-oxidant activity in the presence of iron (5,6), similarly showed elevated levels in most SIDS/SUDI infants with statistically significant increased levels in ALTE when compared with control infants. Mean levels of antioxidants selenium (5,6) and vitamin E (5,6), and vitamin A (with variably reported oxidative effect) (10), were generally low or low normal across all groups compared with adult or recommended levels. When assay levels of these parameters were examined as a function of age, AChE and thyroglobulin in ALTE and control infants increased and decreased respectively over the first 6 months to attain normal adult levels thereafter. Higher levels of iron and ferritin over the first 6 months of life intersected with vitamin C levels, at their height around 4 months of age. There were trends for maternal iron and vitamin supplementation during pregnancy to increase reported levels of these parameters in SIDS infants, and for infant vitamin supplementation using Vitadol C to increase vitamin C levels in supplemented infants. Higher levels of smoking and recreational drug use in mothers of SIDS infants, opioid use within the SIDS group, and compromised sleep situation, did not appear to influence these biochemical parameters per se, but may have contributed to cholinergic, hypoxic, and oxidative stress mechanisms. Likewise GOR and upper respiratory symptoms, more prevalent in ALTE and SIDS infants, showed no apparent relationship with levels of biochemical parameters, although a cholinergic effect through vagal reflexes was possible (15).

Our results support the hypothesis that increased cholinergic tone and oxidative stress effect contribute to the vulnerability of SIDS infants and their age of risk. Exogenous stressors, as discussed, may disturb homeostatic balance when oxidative stress overwhelms antioxidant protection, e.g. with maternal smoking, hypoxia; when cholinergic transmission is increased per AChE inhibition/nicotine stimulation prior to receptor down-regulation [observed with muscarinic receptors (16), although conversely, nicotinic receptors may be up-regulated by nicotine (17)]; during transitions in cholinergic modulation of central respiratory control as a consequence of previous opioid exposure (18), reduced activity of the central muscarinic receptors responsive to hypercapnia and asphyxia (19), or AChE action, direct/indirect, on hydrolysis of opposing respiratory neuropeptides, e.g. substance P (20,21), enkephalin (22); and with mediation of vagal trigger reflexes, e.g. the laryngeal chemoreflex (LCR). The LCR can produce apnoea, laryngeal constriction or closure, and bradycardia, per liquid stimulus to the laryngeal mucosa (15,23). Microaspiration of gastric fluid, laryngeal inflammation, and postnasal drip are putative stimuli for the LCR. This could act as a trigger mechanism in ALTE and SIDS/SUDI infants per the higher rates of GOR, respiratory syncytial virus infection, upper respiratory tract infection/mucus secretion, seen in these infants. The LCR can be further augmented during hypoxia (15), with the use of cholinergic agents (23), and possibly during non-rapid eye movement sleep, when parasympathetic outflow increases and sympathetic outflow decreases (24), reducing opportunity for sympathetic pressor response.

There have been few studies examining these parameters in the context of SIDS. Higher concentrations of blood and serum ferritin were previously reported in SIDS infants compared with post-mortem controls, although this was thought to be characteristic of infant age rather than SIDS specifically (25). A Scandinavian study found an association between increased risk of SIDS and infants not given vitamin A supplementation during the first year of life (26). In other infant studies, higher vitamin C levels in the first few days of life have been associated with an increased risk of dying in very preterm infants (27), and higher thyroglobulin levels in cord blood were observed with combined maternal/paternal smoking during pregnancy, high levels persisting for at least a year, if exposure to passive smoking from both parents was continued (28).

It should be acknowledged that this study lacked sufficient power to draw conclusions about maternal and infant supplementation. The fact, however, that vitamin C can be actively transported across the placenta (29), and oral iron transfer to the foetus up-regulated in women with depleted iron stores (30), suggests that maternal supplementation in pregnancy holds relevance for the foetus and potentially the infant.

Local practice has seen a reduction in iron prophylaxis for pregnant women and an increasing use of over the counter multivitamin preparations. Iron and vitamin supplementation in infants is usually by medical prescription and restricted to low birth weight and premature infants. For these infants, initial vitamin supplementation with Vitadol C and Micelle E (Natural Bio Pty Ltd., Warriewood, Australia) (d-alpha-tocopheryl acetate) may be commenced soon after birth with additional iron supplementation at 4–6 weeks (if not on formula), combined iron and Vitadol C continued thereafter until solids are established (around 6 months corrected).

Our capacity, in this study, to assess the SIDS biochemistry and effect of post-mortem artefact, was also limited given the paucity of post-mortem control data. Ferritin is rapidly released into the blood post-mortem (25), and it is possible that the very high vitamin C levels in SIDS/SUDI infants are simply a marker of significant cerebral insult and release of the vitamin from ascorbate-rich brain tissue (27).

Sudden infant death syndrome nevertheless occurs when tissue iron levels are higher than at any other time because of the high rate of haemoglobin breakdown and relatively low rate of synthesis (25). At 6–20 weeks of age, when risk for SIDS is greatest, high levels of vitamin C potentially interact with high levels of iron, to exacerbate oxidative stress at a time when low levels of selenium and vitamins E and A compromise antioxidant protection. Reduced smoking exposure, and safe sleeping practices may have curbed some cholinergic and oxidative stress effect and decreased SIDS risk accordingly. Further investigation is needed to evaluate best practice for supplementation, in particular with iron and vitamin C, during pregnancy and the first 6 months of life.
